# Effects of chronic exposure to sodium arsenite on hypothalamo-pituitary-testicular activities in adult rats: possible an estrogenic mode of action

**DOI:** 10.1186/1477-7827-4-9

**Published:** 2006-02-16

**Authors:** Kuladip Jana, Subarna Jana, Prabhat Kumar Samanta

**Affiliations:** 1 Department of veterinary Surgery and radiology, west Bengal University of Animal and Fishery Sciences, 37 and 68, K. B. Sarani, Calcutta- 700 037, West Bengal, India; 2 Institute of Molecular Medicine, Bengal Intelligent Park Ltd., Sector-V, Salt Lake Electronics Complex, Calcutta-700 091, India

## Abstract

**Background:**

Inorganic arsenic is a major water pollutant and a known human carcinogen that has a suppressive influence on spermatogenesis and androgenesis in male reproductive system. However, the actual molecular events resulting in male reproductive dysfunctions from exposure to arsenic remain unclear. In this context, we evaluated the mode of action of chronic oral exposure of sodium arsenite on hypothalamo-pituitary- testicular activities in mature male albino rats.

**Methods:**

The effect of chronic oral exposure to sodium arsenite (5 mg/kg body weight/day) via drinking water without or with hCG (5 I.U./kg body weight/day) and oestradiol (25 micrograms oestradiol 3-benzoate suspended in 0.25 ml olive oil/rat/day) co-treatments for 6 days a week for 4 weeks (about the duration of two spermatogenic cycle) was evaluated in adult male rats. Changes in paired testicular weights, quantitative study of different varieties of germ cells at stage VII of spermatogenic cycle, epididymal sperm count, circulatory concentrations of hormones (LH, FSH, testosterone and corticosterone), testicular activities of delta 5, 3beta-hydroxysteroid dehydrogenase (delta 5, 3beta-HSD), 17 beta-hydroxysteroid dehydrogenase (17 beta-HSD), sorbitol dehydrogenase (SDH), acid phosphatase (ACP), alkaline phosphatase (ALP), and lactate dehydrogenase (LDH), as well as the levels of biogenic amines (dopamine, noradrenaline and 5-hydroxytryptamine (5-HT)) in the hypothalamus and pituitary were monitored in this study. Hormones were assayed by radioimmuno- assay or enzyme- linked immunosorbent assay and the enzymes were estimated after spectrophotometry as well as the biogenic amines by HPLC electrochemistry.

**Results:**

Sodium arsenite treatment resulted in: decreased paired testicular weights; epididymal sperm count; plasma LH, FSH, testosterone and testicular testosterone concentrations; and increased plasma concentration of corticosterone. Testicular enzymes such as delta 5, 3 beta-HSD, 17 beta-HSD, and sorbitol dehydrogenase (SDH) were significantly decreased, but those of acid phosphatase (ACP), alkaline phosphatase (ALP), and lactate dehydrogenase (LDH) were significantly increased. A decrease in dopamine or an increase in noradrenaline and 5-HT in hypothalamus and pituitary were also noted after arsenic exposure. Histological evaluation revealed extensive degeneration of different varieties of germ cells at stage VII of spermatogenic cycle in arsenic exposed rats. Administration of human chorionic gonadotrophin (hCG) along with sodium arsenite partially prevented the degeneration of germ cells and enhanced paired testicular weights, epididymal sperm count, plasma and intratesticular testosterone concentrations, activities of delta 5, 3beta-HSD, 17 beta-HSD and sorbitol dehydrogenase along with diminution in the activities of ACP, ALP and LDH. Since many of the observed arsenic effects could be enhanced by oestradiol, it is suggested that arsenic might somehow acts through an estrogenic mode of action.

**Conclusion:**

The results indicate that arsenic causes testicular toxicity by germ cell degeneration and inhibits androgen production in adult male rats probably by affecting pituitary gonadotrophins. Estradiol treatment has been associated with similar effects on pituitary testicular axis supporting the hypothesis that arsenite might somehow act through an estrogenic mode of action.

## Background

Arsenic, as trivalent arsenite (As^3+^) or pentavalent arsenate (As^5+^), is naturally occurring and ubiquitously present in the environment. Arsenical compounds are environmental toxins with multiple effects in animal and human populations [1,2]. Humans are exposed to arsenic mainly through oral or inhalation routs. Oral exposure occurs via consumption of contaminated water, food, and drugs, and exposure can be life long. Occupational exposure, on the other hand, occurs mainly through inhalation via nonferrous ore smelting, semiconductor and glass manufacturing, or power generation by the burning of arsenic contaminated coal [1]. The main source of environmental arsenic exposure in most populations is the drinking water in which inorganic form of arsenic predominate [3,4]. Clearly assessing the risk from, exposure to inorganic arsenic in water supplies is a key issue facing the scientific community. High levels of arsenic in the drinking water can be found in areas within many countries including Taiwan, China, Chile, Mexico, Argentina, Thailand, Finland, Hungary and Bangladesh [4-6], but it is becoming evident that even the low levels of arsenic typically found in India may pose a significant health risk to the humans [5,6]. The frequent uses of arsenic as herbicides, insecticides, rodenticides, food preservatives, and byproduct of used fossil fuel [7] those are challenging the aquatic environment. Although, chronic dermal toxicity, nephrotoxicity, and skin cancer all occurs with arsenic exposure [6]. Arsenic is a multi-site carcinogen in humans, causing tumors in a variety of tissues including lung, skin, and bladder [2,6]. Other studies indicate that the kidney, liver, uterus and prostate may also be target sites of arsenic carcinogenesis in humans [6,8]. Recently arsenic intoxication in experimental animals has been associated with hepatic tumors [8], the inhibition of ovarian steroidogenic function and gonadotrophins secretion [9]. Arsenic exposure also has been associated with an elevation of adrenocortical steroidogenesis and plasma corticosterone level [10], as well as with severe metabolic disorders such as diabetes in humans [11]. Acute arsenic exposure may cause gastrointestinal tract disorders [12], whereas chronic exposure may exert degenerative, inflammatory, and neoplastic changes of the respiratory, heamatopoetic, cardiovascular, and nervous systems [13]. The effect of sodium arsenite on male reproductive system is not well established, although there are some reports in which arsenite intoxication is associated with spermatotoxicity [2,14], inhibition of testicular androgenesis and reduction of the weight of the testes and accessory sex organs [15] in experimental animals. However, the actual molecular events resulting in male reproductive toxicity from exposure of inorganic arsenic remain unclear. There are several possible mechanisms for the anti-gonadal activities of different chemicals. They may exert a direct inhibitory action on the testis; they may affect pituitary causing changes in gonadotrophins concentrations and thus spermatogenic impairment. hCG has a stimulatory effect on the animals own pituitary to secrete FSH, while possessing LH like activity itself [16]. In the present study, changes in enzymatic and hormonal milieu after arsenic with or without hCG treatment were investigated. Repeated arsenic exposure was associated with proliferative pre-neoplastic lesions of the testis [8]. Estrogen treatment has also been associated with proliferative lesions of the testis, disrupts spermatogenesis and inhibits androgen production along with suppressed gonadotrophins secretion from pituitary [17,18]. So, in this study we like to investigate whether the arsenic may act through an estrogenic mode of action on hypothalamo-pituitary- testicular axis.

## Materials and methods

### Animal selection and care

Thirty, Sprague-Dawley (90 days of age), adult male albino rats, weighing 200–250 gm were obtained from the animal facility of the Institute. The animals were housed single per cage under controlled condition of ambient temperature (22 ± 2°C), humidity (60 ± 5%) and photoperiod controlled room (light: dark: 14 h: 10 h) with free access to standard laboratory food (formulated & prepared in our laboratory) and water *ad libitum*. The *NIH Guide for the Care and Use of Laboratory Animals *(NIH publication No. 85 -23 revised 1985: US Department of Health, Education and Welfare, Bethesda, Maryland, USA) was followed throughout the experimental duration. The experimental protocol also met the *National Guidelines on the Proper Care and Use of Animals in Laboratory Research *(Indian Science Academy, New Delhi, India) and was duly approved by the *Animal Ethics Committee of the Institute*.

### Drug treatments

Sodium arsenite were obtained from Sigma Chemical Co. (St. Louis, MO). All the animals were divided equally into 5 groups, with 6 animals per group and their initial body weight were recorded along with a record of their daily water consumption (about 10–12 ml/ day). Animals of each group treated as follows. Group-1 (control rats) orally received 10 ml of arsenic free distilled water as a vehicle. Group-2 (arsenic-treated rats) was given orally 10 ml of water containing sodium arsenite at the dose of 5 mg per kg body weight per day. Group-3 (hCG supplemented, arsenic-treated rats) received in addition to oral exposure of arsenic, subcutaneous injection of hCG at a dose of 5 I.U./kg body weight/day. Group-4 (oestradiol treated rats) received intramuscular injection of 25 μg oestradiol 3-benzoate (Sigma Chemical Co., St. Louis, MO), suspended in 0.25 ml olive oil/rat/day. Group-5 (oestradiol supplemented, arsenic-treated rats) received in addition to oral exposure of arsenic, intramuscular injection of 25 μg oestradiol 3-benzoate suspended in 0.25 ml olive oil/rat/day. All the treatments were continued for 6 days a week for 4 weeks (about the duration of two spermatogenic cycle). The selection of arsenic dose and procedure of administration were based on the prior study [17,19]. The arsenic dose is somehow higher the levels found in drinking water in wide areas of India and other countries where this trace element is present in the range above the admissible limit of 0.01 ppm, according to World Health Organization [5,9]. The doses of hCG and oestradiol were also chosen on the basis of previous studies [16,17].

### Animal sacrifice, collection of blood, brain and reproductive organs

At 1 day after the last day of treatment, all the animals were sacrificed under light ether aesthesia within 8:00 and 10:00 h to avoid any diurnal fluctuation in the concentrations of hormones and neurotransmitters. Care was also taken not to increase in ether inhalation, to prevent any interference with neurotransmitters. Body weight was recorded before and after the treatment. Blood was collected from each animal from dorsal aorta using heparinized syringe (23 gauge needle). Plasma samples were separated by centrifugation frozen and stored at -20°C until all the samples had been collected for the determination of the different hormonal parameters. The paired testes, epididymis, pituitary and hypothalamus were dissected out quickly and washed in 0.9 % (w/v) cold normal saline, pat dried and the wet weight taken in an electrical monopan balance. One of the testis was fixed in Bouin,s fixative for histological evaluation and the other testis was used for the enzyme assays.

### Quantitative study of spermatogenesis

The testis was fixed in Bouin's fixative and embedded in paraffin wax. Sections of 5 μm thickness were taken from the mid portion of each testis and stained with periodic acid Schiff (PAS)-hematoxylin and examined under a light microscope. Quantitative study of spermatogenesis was carried out by counting the relative number of each variety of germ cells at stage VII of the seminiferous cycle, i.e. type-A spermatogonia (Asg), preleptotine spermatocytes (pLSc), mid pachytene spermatocytes (mPSc) and stage 7 spermatids (7Sd), according to the method of Leblond and Clermont [20] and modified by Clermont and Morgentaler [21]. The nuclei of different germ cells (except step 19 spermatids which can not be enumerated precisely) were counted in 20 round tubules in rat. All the counts (crude counts) of the germ cells were corrected for differences in the nuclear diameter by the formula of: true count = (crude count × section thickness)/(section thickness - nuclear diameter of germ cell) [17]. The nuclear diameter of each variety of germ cell was measured with a Leitz micrometer. The possibility of variable tubular shrinkage in the sections of both arsenic and vehicle injected groups were eliminated by the index of tubular shrinkage which was obtained from the average number of Sertoli cell nuclei containing prominent nucleoli in the sections of the treated rats divided by that of the controls [21]. Stage VII spermatogenesis was analyzed because this stage is highly susceptible to testosterone deficiency [22] and also reflects the final stages of spermatid maturation and thus provides evidence of spermatogenesis as a whole [23].

### Epididymal sperm count

Sperms were collected from an equal length of the caudae of the excised epididymis of each rat by flushing with same volume (10 ml) of suspension medium containing 140 mmol NaCl, 0.3 mmol Kcl, 0.8 mmol Na_2_HPO_4_, 0.2 mmol KH_2_PO_4 _and 1.5 mmol D-glucose (pH adjusted to 7.3 by adding 0.1 (N) NaOH) (E Merck). Collected sample was centrifuged at 100 × g for 2 min, and the precipitate part was re-suspended in 10 ml of fresh suspension medium. A fraction of suspension (100 μl) was mixed with an equal volume of 1% Trypan blue in the same medium, and numbers of sperms were counted in four chambers (used for counting of white blood cells) of the hemocytometer slide [24]. At this concentration of Trypan blue (0.5%), the dye was completely excluded by intact sperms, which appeared bright and colorless, but taken up by dead and damaged sperms, which showed blue heads [25]. The sperms number was expressed per ml of suspension.

### Assay of plasma LH, and FSH concentrations

The LH, and FSH concentrations in the plasma were measured using a double antibody radio-immuno-assay (RIA). The plasma concentrations of LH and FSH were measured according to the standard methods [26], with reagents supplied by the Rat Pituitary Distribution Programme and NIDDK (National Institute of Diabetes and Digestive Kidney Diseases, Bethesda, MD, USA). Highly purified rat LH (rLH-I-4) and rat FSH (rFSH-I-8) were iodinated with 1 mCi ^125^I (Bhaba Atomic Research Centre, Mumbai, India), and with freshly prepared chloramine T (Sigma, St.Louis, MO, USA) [27]. Goat antirabbit γ globulin was used as the second antibody (Indo-Medicine, TX, USA). NIDDK- rLH-RP-3 was used as a standard and NIDDK- anti- rLH-S-5 was used for the LH assay. The limit of the detection of LH was 0.05 ng at 80 %. For the FSH, NIDDK- rFSH – RP-2 was used as a standard and NIDDK- anti- rFSH – S-11 was used for the assay. The limit of the detection of FSH was 0.04 ng at 98 %. All the samples were assayed on the same day to avoid the inter assay variation. Intra assay coefficient of variation of LH and FSH assays were 3.5 %.

### Assay of plasma and intra- testicular testosterone concentrations

The testis was homogenized in 0.5 ml of water using Teflon homogenizer that would be fitted into a microfuge tube chilled in ice. Each sample was centrifuged at 10 000 × g for 10 min. The supernatant was removed, frozen, and stored until the hormone assay [28]. Plasma and intra testicular concentrations of testosterone was measured with commercially available kit (IBL, Hamburg, Germany), following the immunoenzymatic method in ELISA reader (Merck, Japan) and according to the standard protocol given by National Institute of Health and Family Welfare (NIHFW, New Delhi, India) [29]. Horseradish peroxidase was used as an enzyme-labeled antigen that made a competition with unlabelled antigen for binding with a limited number of antibody sites on the micro- plates (solid phase). Each testosterone concentration was calculated from a standard curve with 5 standards. The absorbance of standard and sample was monitored against the blank at 450 nm. The cross-reaction of the testosterone antibody to dehydrotestosterone was 10% and intra-run precision had a coefficient of variation of 6.2%. All the samples were included in a single assay. The assay validated in respect to correctness of the data in our laboratory was 98%.

### Estimation of plasma concentration of corticosterone

Plasma concentration of corticosterone was measured by spectroflurometrically (F-3010, Hitachi, Japan) according to the method of Glic et al. [30] and modified by Silber [31]. The fluorescence was measured at 463 nm (excitation) and 518-nm (emission) by setting the instrument at a spectroflurometric reading 80, with a standard corticosterone (Sigma) solution (1.6 μg/ml). A minimum of 1.6 μg corticosterone/100 ml plasma can be measured by this method. The concentration of corticosterone was expressed as μg/100 ml of plasma.

### Assay of testicular key androgenic enzyme activities

The testicular Δ^5^, 3β-HSD activity was measured according to the method of Talalay [32]. The testicular tissue of each animal was homogenized in 15% spectroscopic grade glycerol (BDH, Mumbai, India) containing 5 mmol potassium phosphate (Loba, Mumbai, India) and 1 mmol EDTA (Organon, Calcutta, India.) at a tissue concentration of 100 mg/ml. The homogenizing mixture was centrifuged at 10,000 × g for 30 min at 4°C. The supernatant (1 ml) was mixed with 1 ml of 100 μmol sodium pyrophosphate buffer (pH 8.9) and 30 μg of dehydroepiendrosterone (Sigma) in 40 μl of ethanol and 960 μl of 25% BSA (Sigma), making the incubation mixture a total of 3 ml. The enzyme activity was measured after addition of 0.5 μmol of NAD (Sigma) to the tissue supernatant mixture in a U2000 spectrophotometer (Hitachi, Japan) cuvette at 340 nm against a blank (without NAD). One unit of enzyme activity is the amount causing a change in absorbence of 0.001/min at 340 nm.

The activity of testicular 17β-HSD was measured biochemically [33] using the same supernatant prepared for the assay of Δ^5^, 3β-HSD (above). The supernatant (1 ml) was mixed with 1 ml of 440 μmol sodium pyrophosphate buffer (pH 10.2), 40 μl of ethanol containing 0.3 μmol of testosterone (Sigma) and 960 μl of 25% BSA (Sigma), making the incubation mixture a total of 3 ml. The enzyme activity was measured after addition of 1.1 μmol NAD (Sigma) to the tissue supernatant mixture in a U2000 spectrophotometer cuvette at 340 nm against a blank (without NAD). One unit of enzyme activity is equivalent to a change in absorbance of 0.001/min at 340 nm.

### Assay of testicular acid phosphatase (ACP) and alkaline phosphatase(ALP) activities

The activity of testicular acid phosphatase was measured following the standard method of Vanha-Perttula and Nikkanen [34]. Testicular tissue was homogenized in 0.02 mol Tris HCl buffer, pH 7.5, at a tissue concentration of 10 mg/ml. 0.25 ml of a tissue homogenate was added in a centrifuge tube containing 1 ml buffer (1.0 ml p- nitrophenol phosphate (PNPP) in 0.1 mol acetate buffer, pH 5.0). The mixture was incubated at 37°C for 30 min in a water bath. Then the reaction was terminated by addition of 0.1 ml 0.1 mol NaOH. The assay was based on the formation of p- nitrophenol (PNP) in the hydrolysis of PNPP. The activity was measured spectrophotometrically at 420 nm using U2000 spectrophotometer. The concentration of the sample was obtained from a standard curve and expressed as μg of PNP liberated/ mg of tissue/ hr. For determination of alkaline phosphatase the same homogenizing media was used and the tissue concentration was also same. 0.25 ml of a tissue homogenate was added in a centrifuge tube containing 1 ml buffer (1 mmol PNPP in 1 mol Tris buffer, pH- 8.0). The mixture was incubated at 37°C for 30 min in a water bath. Then the reaction was terminated by addition of 0.1 ml 0.1 mol NaOH. The assay was based on the formation of PNP in the hydrolysis of PNPP. The activity was measured spectrophotometrically at 420 nm using U2000 spectrophotometer. The concentration of the sample was obtained from a standard curve and expressed as μg of PNP liberated/ mg of tissue/ hr [35].

### Assay of testicular lactate dehydrogenase (LDH) and sorbitol dehydrogenase (SDH) activities

Testicular lactate dehydrogenase and sorbitol dehydrogenase activities were measured using the kits purchased from Sigma Chemical Co. Testicular tissue was homogenized in ice-cold 0.1 mol Tris EDTA buffer, pH 8.0 at a tissue concentration of 10 mg/ml and then the homogenizing mixture was centrifuged at 10,000 × g for 30 min at 4°C. The supernatant was used for these enzymes estimation. The LDH assay is based on the inter-conversion of lactate and pyruvate and the SDH assay is based on inter- conversion of Fructose and Sorbitol. During the reduction of pyruvate or fructose, an equimolar amount of NADH is oxidized to NAD. The oxidation of NADH results in a decrease in absorbance at 340 nm. The rate of decrease of absorbance at 340 nm is directly proportional to LDH or SDH activity in the sample, where one unit of LDH or SDH activity is defined as 0.1 μmol of substrate transformed/ minute at pH 7.6 at 25°C.

### Estimation of neurotransmitters by HPLC

Pituitary and hypothalamus were weighted and sonicated in 0.1 mol ice-cold perchloric acid/ lit containing 0.05% EDTA. They were centrifuged at 10 000 × g for 10 min at 4°C and 10 μl of the supernatant was injected directly into an HPLC system (Waters, Milford, MA) to determine noradrenaline, dopamine and 5 HT according to the method of Mohanakumar et al. [36]. The HPLC system was equipped with a Universal injector, electrochemical detector (460, Waters) and an ion-pair, Ultrasphere RP analytical column (4.6 cm × 25 cm) with 5 μm particle size (Beckmann, Fullerton, CA). The mobile phase contained 8.65 mmol haptane sulphonic acid/ lit, 0.27 mmol EDTA/ lit, 13% (v/v) acetonitrile, 0.4–0.45% (v/v) triethylamine and 0.20–0.25% (v/v) phosphoric acid. The flow rate was 0.7 ml/min and the electro- detection was performed at 0.74 V. The data were collected and integrated in a Waters 745B data module. The peak heights were measured and compared with authentic samples. Results are presented as pmole/ mg fresh tissue and are uncorrected values.

#### Statistical analysis

Results of the experiment were expressed as mean and standard error of mean of different groups. The differences between the mean values were evaluated by ANOVA followed by multiple student's t- test [37]. The values for p < 0.01 were considered significant. Accordingly, a statistical software package (SPSS) was used.

## Results

### Food consumption and body growth and relative organ weight

No differences in food consumption were seen in any of the group of animals throughout the experimental schedule. In all the treated groups, the body weight was not significantly different from that of controls. The paired testicular weights were significantly (p < 0.01) decreased after arsenic or oestradiol treatments with respect to control indicating the testicular atrophy and damage. Co- administration of hCG and arsenic restored this relative organ weights towards control level. Co- administration of oestradiol with arsenic showed more diminution in this parameter in comparison to the controls (Table 1).

**Table 1:** Testicular weights, quantitative analysis of spermatogenesis at stage VII and epididymal sperm counts in different experimental groups of adult male rats.

Treatment	Paired testicular weight (g)	**Spermatogenesis at stage VII**				Epididymal sperm count (no/ml)
			
		ASg	pLSc	mPSc	7Sd	
Control	2.31 ± 0.11	1.32 ± 0.21	18.62 ± 2.12	22.16 ± 1.62	64.30 ± 2.54	10260 ± 1410
Arsenic	1.74 ± 0.08*	0.65 ± 0.08*	10.54 ± 1.25*	12.20 ± 0.76*	30.28 ± 2.15*	6350 ± 308*
Arsenic + hCG	2.10 ± 0.05	1.48 ± 0.16	15.16 ± 1.82*	18.62 ± 0.64*	46.84 ± 3.24*	8940 ± 246*
Oestradiol	1.87 ± 0.06*	0.72 ± 0.12*	12.10 ± 1.12*	14.52 ± 0.42*	32.46 ± 1.31*	7105 ± 415*
Arsenic + Oestradiol	1.65 ± 0.09*	0.42 ± 0.05**	10.14 ± 1.21*	10.56 ± 1.32*	25.65 ± 1.42**	5120 ± 314*

Each value represents mean ± SE, n = 12. (ANOVA followed by multiple comparison two-tail t-tests, * p < 0.01, ** p < 0.001 as compared with respective control). Asg = spermatogonia-A, pLSc = preleptotine spermatocytes, mPSc = midpachytene spermatoeytes, 7Sd = stage 7 spermatids.

### Effect on spermatogenesis

Quantitative study of spermatogenesis at stage VII revealed detrimental effect of sodium arsenite treatment. A significant reduction in the numbers of Asg, pLSc, mPSc and 7Sd at stage VII of the seminiferous epithelium cycle was observed after arsenic or oestradiol treatment when compared to the controls. Co- administration of hCG and arsenic increased the numbers of Asg, pLSc, mPSc and 7Sd in comparison with arsenic- treated rats but a significant reduction in the numbers of pLSc and mPSc was still observed when compared with corresponding vehicle injected controls. Administration of hCG prevented the degeneration of 7Sd, although the count showed a lower value when compared with vehicle injected controls. Furthermore, the inhibitory effect of arsenic on spermatogenesis was enhanced by co- administration of oestradiol and arsenic, as the degeneration in different generations of germ cells was more drastic in this group with respect to the group of animals treated with only arsenic (group-2). Chronic exposure to sodium arsenite as well as oestradiol resulted in a testicular damage, which showed reduction in the number of germ cells from the seminiferous tubules (Table 1).

### Effect on epididymal sperm count

Sperm counts also revealed a significant (p < 0.01) reduction in the number of spermatozoa after both sodium arsenite or oestradiol treatment when compared with respective vehicle treated rats. Co- administration of hCG and arsenic enhanced the number of epididymal spermatozoids in comparison with arsenic-treated rats, though the number did not reach the control level. Moreover, the co- administration of oestradiol and arsenic enhanced the antispermatogenic effect observed in rats treated only with arsenic (group-2) or oestradiol (group-4) (Table 1).

### Effect on testicular Δ^5^, 3β-HSD, 17β-HSD and SDH activities

**Figure 1:**
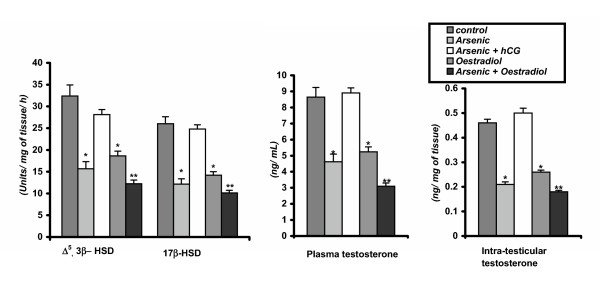
Testicular androgenic key enzyme activities, plasma and intra-testicular testosterone concentrations in control and different experimental rats. Data as mean + SE, n = 12, ANOVA followed by multiple comparison two- tail t- test (* p < 0.01, ** p < 0.001 as compared with respective control.)

**Figure 3:**
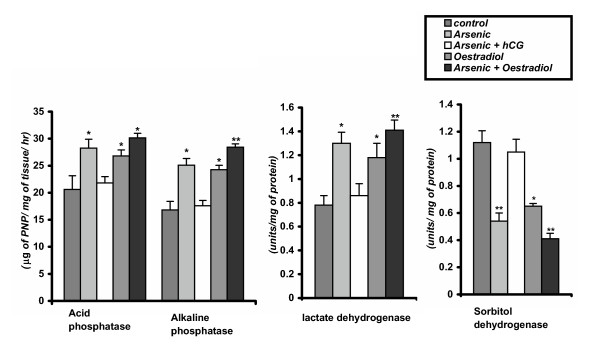
Testicular acid phosphatase, alkaline phosphatase, lactate dyhydrogenase and sorbitol dehydrogenase enzymes activities in control and different experimental mature male rats. Data as mean + SE, n = 12, ANOVA followed by multiple comparison two- tail t- test (* p < 0.01, ** p < 0.001 as compared wih respective control.)

Testicular Δ^5^, 3β-HSD and 17β-HSD are the key enzymes for testosterone biosynthesis. The inhibitory responses from sodium arsenite or oestradiol treatments were noted in testicular Δ^5^, 3β-HSD or 17β-HSD or SDH activities. The activities of Δ^5^, 3β-HSD, 17β-HSD and SDH in testicular tissue were decreased significantly (p < 0.01) in both the arsenic or oestradiol treated groups in comparison to the vehicle control group. Moreover, co- administration of hCG and arsenic restored these enzyme activities to the control level. Co- administration of oestradiol and arsenic enhanced the inhibitory responses on these testicular enzyme activities observed in rats treated only with arsenic (group2) or oestradiol (group 4) (Figure 1&3).

### Effect on plasma concentrations of LH and FSH

**Figure 2:**
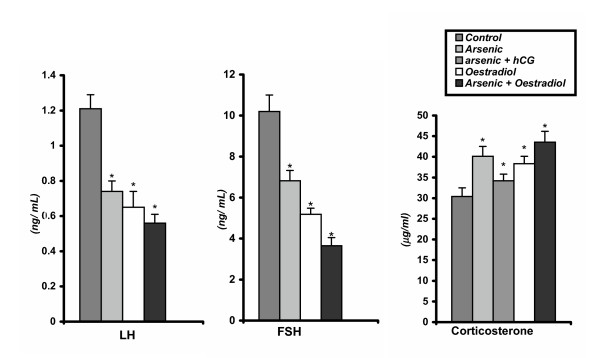
Plasma LH, FSH and corticosterone concentrations in control and different experimental rats. Data as mean + SE, n = 12, ANOVA followed by multiple comparison two- tail t- test. (* p < 0.01, ** p < 0.001 as compared with respective control.).

Chronic arsenic or oestrdiol treatment significantly (p < 0.01) diminished the plasma LH and FSH concentrations. Moreover, the co- administration of arsenic and oestradiol revealed greater diminution in these hormone concentrations observed in rats treated only with arsenic (group-2) or oestradiol (group-4)(Figure 2).

### Effect on plasma testosterone, corticosterone and intratesticular testosterone concentrations

Chronic exposure of sodium arsenite or oestradiol significantly (p < 0.01) decreased plasma and intratesticular testosterone concentrations. Co- administration of hCG and arsenic increased the testicular testosterone and plasma testosterone concentrations above those of the vehicle injected control rats. Furthermore, the inhibitory effect arsenic on this hormone concentration was enhanced by co-administration of oestradiol with arsenic (group-5) (Figure 1). There was a significant elevation in plasma concentrations of corticosterone at any of the group treated with arsenic or oestradiol in comparison to the vehicle control group, though there was no significant difference in this hormone level in both the group of animals that treated with only oestradiol or in combined treatment of oestradiol and sodium arsenite. After co- administration of hCG and arsenic significantly diminished this hormone concentration in comparison with arsenic-treated rats but still greater than the control level (Figure 2).

### Effect on testicular ACP, ALP, and LDH activities

The testicular activities of ACP, ALP and LDH were significantly (p < 0.01) increased after sodium arsenite or oestradiol treatment in comparison to the vehicle control, which reflects the testicular degeneration and cytotoxicity. Co- administration of hCG and arsenic significantly decreased these enzyme activities to the control level indicating significant protection of germinal epithelium from arsenic toxicity. Moreover, co- administration of oestradiol and arsenic revealed more elevation in these enzyme activities in respect to the rats exposed to only with arsenic (Figure 3).

### Effect on neurotransmitters in hypothalamus and pituitary

**Figure 4:**
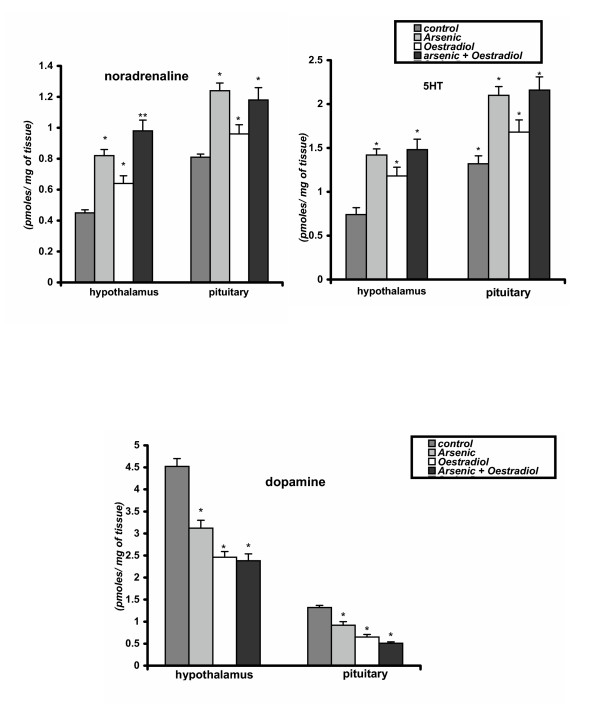
a. Levels of noradrenaline and 5HT in hypothalamus and pituitary in control and different experimental groups of mature male albino rats. Data as mean + SE, n = 12, ANOVA followed by multiple comparison two- tail t- test. (* p < 0.01, ** p < 0.001 as compared with respective control.) b. The levels of dopamine in hypothalamus and pituitary in control and different experimental groups of mature male albino rats. Data as mean + SE, n = 12, ANOVA followed by multiple comparison two- tail t- test. (* p < 0.01, ** p < 0.001 as compared with respective control.)

Chronic oral sodium arsenite treatment resulted in a significant decrease in dopamine and also a significant increase in noradrenaline and 5-HT in both the hypothalamus and pituitary (p < 0.01). These findings may indicate the effect of arsenic on hypothalamo- pituitary axis through the modulation in the levels of brain neurotransmitters by the stimulation of adrenergic and serotonergic neurons or by inhibition of dopaminargic neuronal activities. Oestradiol treatment associated with the effects similar to those induced by arsenic exposure. Moreover, the co-administration with oestradiol and arsenic enhanced the effects observed in rats treated only with arsenic (group-2) (Figure 4a&4b).

## Discussion

This study is first to show that chronic oral exposure of a known environmental toxicant and human carcinogen, arsenic, to adult male rats can alter reproductive functions by decreasing the paired testicular mass, inhibiting testicular androgenesis and decreasing concentrations of testosterone and gonadotrophins along with increasing adrenocortical activity. Paired testicular mass, a valuable index of reproductive toxicity in male animals [38] decreased in arsenic-treated animals and this decrease in testicular mass was consistent with elimination of germ cells [39]. Similar effects were also observed after treatment with oestradiol alone or in combination with arsenic. This antigonadal activity of oestradiol is in agreement with the previous findings [18,40-43].

Testicular steroidogenic events, Δ^5^, 3β-HSD and 17β-HSD play a key regulatory role, as these are the prime enzymes in testicular androgenesis [23,44]. The diminution in these enzymes by sodium arsenite treatment in our study is in agreement with the findings of others, where arsenic treatment was associated with the inhibition of testicular androgenesis [15]. A dose-dependent decrease in plasma and intratesticular concentrations of testosterone in arsenic-treated rats may occur due to the inhibition of these testicular androgenic enzymes activities, because these enzymes are responsible for the regulation of testosterone biosynthesis [23,45]. Moreover, the inhibition of testicular androgenic enzymes in arsenic-treated rats may be a result of low plasma levels of LH as this is a prime regulator of testicular androgenic enzymes activities [46,47]. In the present study, the spermatogenic disorder has been reflected by the diminution in the number of different generations of germ cells at stage VII of spermatogenic cycle. These findings also corroborated with the previous findings with arsenic in testis [15]. The inhibition in spermatogenesis is may be due to low level of gonadotrophins i.e. LH and FSH [48]. LH and FSH are required for quantitatively normal spermatogenesis in pubertal rats [22,49]. The reduction of LH and FSH and consequent reduction in testosterone production may therefore, be held responsible for the arsenic induced changes in spermatogenesis. This prediction for low plasma concentration of LH, FSH and testosterone in arsenic-treated rats are consistent with our results when plasma concentrations of LH, FSH and testosterone have been decreased in treated rats in a dose-dependent manner. Moreover, the reduction in the number of Asg in arsenic-treated rats is possibly due to the low level of FSH, as FSH inhibits the normal degeneration of Asg and reduced FSH secretion may promotes Asg degeneration [15,48]. Besides this, the germ cell degeneration by arsenic treatment is may be due to low intratesticular concentrations of testosterone, as high level of testosterone in testis is essential for normal spermatogenesis as well as for the maintenance of structural morphology and normal physiology of seminiferous tubule [50,51]. However the maturation of mPSc and 7Sd are testosterone dependent [48]. The reduction in the number of mPSc and 7Sd following sodium arsenite treatment is due to diminution in testosterone production. In experimental circumstances in which the intratesticular testosterone concentration is reduced a significant germ cell death is seen [52]. Testosterone is required for the attachment of different generations of germ cells in seminiferous tubules and therefore low level of intratesticular testosterone may lead to detachment of germ cells from seminiferous epithelium and may initiate germ cell apoptosis [53]. The decreases in plasma testosterone concentrations may be explained by the fact that the arsenic treatment causes a decrease in plasma LH concentrations. In male rats, circulating LH is responsible for maintaining normal plasma testosterone concentrations [54]. The possibility of low plasma level of gonadotrophins in this experiment may be due to elevated secretion of glucocorticoids from adrenal gland as the high level of corticosterone also observed in arsenic-treated animals. Moreover, arsenic activates the stress signal pathway, the hypophysial- adrenocortical axis and increased the secretion of ACTH from pituitary [55]. The elevation of plasma levels of corticosterone, which may suppress the sensitivity of gonadotroph cells to GnRH and therefore may, prevents gonadotrophin secretion [56]. The high levels of ACTH and corticosterone also directly suppress testosterone production and secretion by decreasing the testicular LH receptor [57] resulting the reduction of spermatogenesis and epididymal sperm count. The diminution in the epididymal sperm count in arsenic-treated animals is due to the lower concentrations of testosterone as the sperm production in testis and maturation in epididymis is under the control of testosterone [51]. Moreover, the increased activity of testicular acid phosphatase and alkaline phosphatase in arsenic-treated rats also reflects testicular degeneration, which may likely be a consequence of suppressed testosterone and indicative of lytic activity [58]. The activities of SDH and LDH in testicular tissue are associated with the maturation of the germinal epithelial layer of seminiferous tubule [14,59]. The activity of SDH increased markedly through out the maturation of germ cells and is reported to decrease during the depletion of germ cells [59]. Moreover, the activity of this enzyme is regulated by testosterone and the diminution in this enzyme activity after arsenic exposure is due to the decreased production of testosterone [60]. LDH is reported to be present in higher amounts in the testis of newborn rats and its activity declines with the development of the testis. The increase in activity of LDH and decrease in that of SDH observed in treated animals suggest that this chemical exposure causes deterioration of germinal epithelium [14,59,60]. Since the body weight gain was not altered significantly in arsenic-treated rats in comparison to controls. The deleterious effect of arsenic on the male reproductive system may be due to the toxic effect of arsenic itself on this particular system but not to the health of the animals. Sodium arsenite induced diminution in plasma gonadotrophins may be due to the low level of dopamine and elevated levels of noradrenaline and 5-HT in hypothalamus and pituitary as these catacholamines are the important regulators for gonadotrophins secretion and synthesis [61]. However, the diminution in the level of dopamine by arsenic treatment caused the suppressed secretion of gonadotrophins as dopamine has the stimulatory effect on gonadotrophins secretion [19]. Increases in noradrenaline and 5-HT and decrease in dopamine in hypothalamus and pituitary indicate that the effect of the arsenic can be potentiated in an environment in which steroid concentrations are high.

Co- administration of hCG in arsenic-treated rats resulted a significant protection in testicular Δ^5^, 3β-HSD and 17β-HSD activities, plasma and intratesticular testosterone to the control along with increased total sperm count. The protection of steroidogenesis in arsenic-treated animals by hCG is due to the stimulatory effects of hCG on steroidogenesis [62,63]. However, in this experiment, administration of hCG along with arsenic partially prevented the degeneration of 7Sd but failed to restored spermatogenesis quantitatively. Although hCG failed to restore the number of spermatocytes and spermatids, but the numbers of Asg were significantly increased compared with that of vehicle control. Though the effect of hCG on Asg at stage VII of the seminiferous cycle in adult rats is not clear from this experiment but the initiation and maintenance of spermatogenesis require LH and FSH in pre-pubertal and pubertal rats [48] and hCG has a stimulatory effect on the animals own pituitary to secrete FSH, while possessing LH like activity itself [16,64]. LH stimulates Leydig cells to produce testosterone within the testis. Intratesticular testosterone is an absolute prerequisite for normal spermatogenesis [65]. FSH is also vital for normal testicular function and is necessary for quantitatively normal spermatogenesis [66]. Moreover, hCG maintains the baseline levels of intratesticular testosterone in gonadotrophins withdrawal from exogeneous testosterone administration [63]. The hCG acts as a LH and stimulated the Leydig cells to produce testosterone and that maintains the normal spermatogenesis after gonadotrophin suppression [62,63]. The restoration of the activities of SDH, LDH, ACP, and ALP in hCG co- administration may be due to the significant protection of germ cells from arsenic induced degeneration. However, the direct detrimental action of arsenic on germ cells can't be ruled out since administration of hCG along with arsenic treatment failed to restore quantitatively the number of all varieties of germ cells at stage VII of the seminiferous cycle.

The spermatogenic degeneration with arsenic treatment in the present work could also be consistent with an estrogen related mechanism. It is quite clear from this experiment that chronic oestradiol treatment in adult rat can induce the same spermatogenic degeneration as induced by arsenic treatment. Moreover, the antispermatogenic effects of arsenic could be enhanced by oestradiol co- administration in arsenic-treated animals. It is widely accepted that oestradiol treatment mimics the effects of hypophysectomy in the testis, due to gonadotrophins and testosterone suppression [17,18,67]. Oestradiol also suppressed the transcription of gonadotrophin genes [68]. In our study, oestradiol treatment resulted more than 75% suppression of LH, FSH and testosterone concentrations, respectively; that is its effect on germ cells degeneration should be equivalent to other gonadotrophin suppressing treatments [17,42,43]. Moreover, oestradiol exposure has been shown to decrease Leydig cell volume and impair the levels of mRNA and protein of important enzymes of steroidogenesis [69,70] that is consistent with our result where the key steroidogenic enzymes were diminished after oestradiol treatment. Oestradiol treatment resulted severe degeneration of germ cells at stage VII of seminiferous epithelium cycle. Earlier morphological studies showed that withdrawal of either gonadotrophins [67] or testosterone [52] leads to a marked increase in this type of cell death mainly at stage VII. It has been reported that germ cell apoptosis occurs at several stages of the cycle after either gonadotrophin [71] or testosterone [72] deprivation. Blanco- Rodriguez and Martinez-Garcia [18] also showed that oestradiol treatment caused germ cell apoptosis along with suppressed the gonadotrophin and testosterone concentrations in rat.

Several arsenic-treated animals showed reproductive tract abnormalities including gonadotrophins and androgen suppression along with germ cell degeneration in testes, effects similar to those induced by estrogenic agonists, such as di-ethylstilbestrol, methoxychlor and tamoxifen [42]. Further study, demonstrated that the enhanced expression of estrogen receptors (ERs) and various ER- associated gene products were evident in arsenic induced testicular lesions [8] suggesting that arsenate may exhibit the estrogenic properties.

## Conclusion

From the present study, it may be suggested that sodium arsenite treatment impairs male reproductive systems. It appears, therefore, that arsenic treatment produces degenerative changes in the germ cells and inhibits androgen production acting primarily at the level of pituitary to inhibit the release of LH and FSH, that is partially protected by hCG co- administration. Estradiol treatment has been associated with similar effects on pituitary testicular axis and also co- treatment of oestradiol with arsenic could enhance these effects supporting the hypothesis that arsenite might somehow act through an estrogenic mode of action. However, the possible action of arsenic as an environmental estrogen deserves additional study.

## List of abbreviations

ACP, acid phosphatase; ACTH, adrinocorticotrophic hormone; ALP, alkaline phosphatase; Asg, spermatogonia-A; Δ^5^, 3β-HSD, delta 5, 3 beta-hydroxysteroid dehydrogenase; 17β-HSD, 17 beta-hydroxysteroid dehydrogenase; ELISA, enzyme linked immunosorbent assay; RIA, radioimmunoassay; ER, estrogen receptor; FSH, follicle stimulating hormone; GnRH, gonadotrophin releasing hormone; hCG, human chorionic gonadotrophin; HPLC, high pressure liquid chromatography; 5HT, 5 hydroxytryptamine; HPT, hypothalamus-pituitary-testicular axis; ITT, intratesticular testosterone; LDH, lactate dehydrogenase; LH, luteinizing hormone; mPSc, midpachytene spermatoeytes; NE, norepinephrine; NIDDK, National Institute of Diabetes and Digestive Kidney Diseases; pLSc, preleptotine spermatocytes; 7Sd, stage 7 spermatids; SDH, sorbitol dehydrogenase.

## References

[B1] Liu SX, Athar M, Lippai I, Waldren C, Hei TK (2001). Induction of oxyradicals by arsenic: implication for mechanism of genotoxicity. Proc Natl Acad Sci USA.

[B2] Waalkes MP, Ward JM, Liu J, Diwan BA (2003). Transplacental carcinogenicity of inorganic arsenic in the drinking water: induction of hepatic, ovarian, pulmonary, and adrenal tumors in mice. Toxicol Appl Pharmacol.

[B3] Bates MN, Smith AH, Hopenhayn-Rich C (1992). Arsenic ingestion and internal cancers: a review. Am J Epidemiol.

[B4] Pott WA, Bengamin SA, Yang RSH (2001). Pharmacokinetics, metabolism and carcinogenecity of arsenic. Rev Environ Contam Toxicol.

[B5] Chatterjee A, Das D, Mandal BK, Samanta G, Banerjee P (1995). The biggest arsenic calamity in the world. Analyst.

[B6] NRC (1999). Arsenic in the drinking water.

[B7] Flora SJS, Dube SN, Arora U, Kannan GM, Sukla MK, Malhotra PR (1995). Therapeutic potential of meso 2, 3- dimercaptosuccinic acid or 2,3- dimercaptopropane 1- sulfonate in chronic arsenic intoxication in rats. Biomatals.

[B8] Waalkes MP, Keefer LK, Diwan BA (2000). Induction of proliferate lesions of the uterus, testes, and liver in Swiss mice given repeated injections of sodium arsenate: possible estrogenic mode of action. Toxicol Appl Pharmacol.

[B9] Chattopadhyay S, Ghosh S, Chaki S, Debnath J, Ghosh D (1999). Effect of sodium arsenite on plasma levels of gonadotrophins and ovarian steroidogenesis in mature albino rats: duration dependent response. J Toxicol Sci.

[B10] Ghosh D, Chattopadhyay S, Debnath J (1999). Effect of sodium arsenite on adrenocortical activity in immature female rats: Evidence of dose-dependent response. J Environ Sci.

[B11] Tseng CH, Tseng CP, Chiou HY, Hsueh YM, Chong CK, Chen CJ (2002). Epidemiologic evidence of diabetogenic effect of arsenic. Toxicol Lett.

[B12] Goebl HH, Schmidt PF, Bohl J, Teltenborn B, Kramer G, Gutman L (1990). Polyneuropathy due to arsenic intoxication: biopsy studies. J Neurol.

[B13] Naiger RD, Osweiler GD (1989). Effect of sub acute low-level dietary sodium arsenite on dogs. Fundam Appl Toxicol.

[B14] Pant N, Murty RC, Srivastava SP (2004). Male reproductive toxicity of sodium arsenite in mice. Human Exp Toxicol.

[B15] Sarkar M, Ray Chaudhuri G, Chattopadhyay A, Biswas NM (2003). Effect of sodium arsenite on spermatogenesis, plasma gonadotrophins and testosterone in rats. Asian J Androl.

[B16] Ghosh S, Misro M, Das UB, Maiti R, Debnath JM, Ghosh D (2001). Effect of human chorionic gonadotrophin coadministration on ovarian steroidogenic and folliculogenic activities in cyclophosphamide treated albino rats. Repro Toxicol.

[B17] Sarkar R, Mohanakumar KP, Chowdhury M (2000). Effects of an organophosphate pesticide, quinalphos, on the hypothalamo-gonadal axis in adult male rats. J Reprod Fertil.

[B18] Blanco- Rodriguez J, Martinez- Garcia C (1997). Apoptosis pattern by oestradiol treatment of the seminiferous epithelium of the adult rat. J Reprod Fertil.

[B19] Chattopadhyay S, Ghosh S, Chaki S, Ghosh D, Debnath J (2003). Effect of dietary co-administration of sodium selenite on arsenite- induced ovarian and uterine disorders in mature albino rats. Toxicol Sci.

[B20] Leblond CP, Clermont Y (1952). Definition of the stage of the cycle of seminiferous epithelium in the rat. Annals NY Acad Sci.

[B21] Clermont Y, Morgentaler H (1955). Quantitative study of spermatogenesis in hypophysectomized rat. Endocrinology.

[B22] Russell LD, Alger LF, Naquin LG (1987). Hormonal control of pubertal spermatogenesis. Endocrinology.

[B23] Ghosh D, Chowdhury A, Biswas NM, Ghosh PK (1990). Effect of lithium chloride on testicular steroidogenesis and gametogenic functions in male albino rats. Life Sci.

[B24] Alvarez JG, Story BT (1984). Assessment of cell damage caused by spontaneous lipid peroxidation in rabbit spermatozoa. Biol Reprod.

[B25] Samanta L, Chainy GBN (1997). Comparison of hexachloro-cyclohexane induced oxidative stress in the testis of immature and adult rats. Comp Biochem Physiol.

[B26] Moudgal NR, Madhwa Raj HG, Jaffe BM, Behrman HR (1974). Pituitary gonadotrophins. Methods of Hormone Radioimmunoassay.

[B27] Greenwood FO, Hunter WM, Glover JS (1964). The preparation of ^131^I labeled human growth hormone of high specific activity. Biochem J.

[B28] Tohda A, Matsumiya K, Tadokoro Y (2001). Testosterone spermatogenesis in juvenile spermatogonial depletion (jsd) mice. Biol Reprod.

[B29] Srivastava TG (2002). Enzyme linked immunosorbent assay for steroid hormones. Orientation training course on research methodology in reproductive biomedicine.

[B30] Glic D, Dorothy Van R, Lavine S (1964). Fluorometric determination of corticosterone and cortisol in 0.02–0.05 milliliters or sub-milligram samples of adrenal tissue. Endocrinology.

[B31] Silber RH, Glic D (1966). Fluorometric analysis of corticoids. Methods in biochemical analysis.

[B32] Talalay P, Colowick SP, Kaplan NO (1962). Hydroxysteroid dehydrogenase. Methods in Enzymology.

[B33] Jarabak J, Adams JA, Williams-Ashman HG, Talalay P (1962). Purification of a 17β-hydroxysteroid dehydrogenase of human placenta and studies on its transhydrogenase function. J Biol Chem.

[B34] Vanha-Perttula T, Nikkanen V (1973). Acid phosphatases of the rat testis in experimental conditions. Acta Endocrinol.

[B35] Malymy M, Horecker BL (1966). Alkaline phosphatase. Methods in Enzymology Volume IX.

[B36] Mohonakumar KP, deBartelomies A, Wu RM, Yeh KJ, Sternberger LM, Peng SY, Murphy DL, Chiueh CC (1994). Ferrous citrate complex and nigral degeneration: evidence for free radical formation and lipid peroxidation. Annals NY Acad Sci.

[B37] Das D, Das A (1998). Statistics in Biology and Psychology.

[B38] Aman RP (1982). A critical review of methods for evaluation of spermatogenesis from seminal characteristics. J Androl.

[B39] Chapin RE, Lamb JC (1984). Effect of ethylene glycol monoethyl ether on various parameters of testicular function in the F344 rats. Environ Health Perspectives.

[B40] Van der Molen HJ, Brinkmann AO, Jong FH, Romerts FF (1981). Testicular oestrogens. J Endocrinol.

[B41] Smith EP, Boyd JB, Frank GR, Takahashii H, Cohen RM, Specker B, William TC, Lubahn DB, Korach SK (1994). Estrogen resistance caused by a mutation in the estrogen-receptor gene in a man. New Engl J Med.

[B42] Akinbemi BT, Hardy MP (2001). Oestrogenic and antiandrogenic chemicals in the environment: effects on male reproductive health. Ann Med.

[B43] Akinbemi BT (2005). Estrogen regulation of testicular function. Reprod Biol Endocrinol.

[B44] Jana K, Samanta PK (2006). Evaluation of single intratesticular injection of calcium chloride for non-surgical sterilization in adult albino rats. Contraception.

[B45] Jana K, Ghosh D, Samanta PK (2005). Evaluation of single intratesticular injection of calcium chloride for non-surgical sterilization of male goats (*Capra hircus*): a dose-dependent study. Anim Reprod Sci.

[B46] Shaw MJ, Georgapouls LE, Payne AH (1979). Synergistic effect of FSH and LH and testicular Δ^5^, 3β-hydroxysteroid dehydrogenase isomerase. Application of a new method for the separation of testicular compartments. Endocrinology.

[B47] Kerr JB, Sharpe RM (1986). Effects and interaction of LH and LHRH agonist on testicular morphology and function in hypophysectomized rats. J Reprod Fertil.

[B48] Chowdhury AK (1979). Dependence of testicular germ cells on hormones: a quantitative study in hypophysectomized testosterone treated rats. J Androl.

[B49] Kulin HE, Reiter EO (1973). Gonadotrophins during childhood and adolescence: a review. Pediatrics.

[B50] Sharpe RM, Donachie k, Cooper I (1988). Re-evaluation of the intratesticular level of testosterone required for quantitative maintenance of spermatogenesis in the rat. J Endocrinol.

[B51] Sharpe RM, Maddocks S, Millar M, Saunders PTK, Kerr JB, Mckinnell C (1992). Testosterone and spermatogenesis: identification of stage dependent, androgen- regulated proteins secreted by adult rat seminiferous tubules. J Androl.

[B52] Kim JM, Ghosh SR, Weil ACP, Zirkin BR (2001). Caspase- 3 and caspase- activated dioxyribonuclease are associated with testicular germ cell apoptosis resulting from reduced intratesticular testosterone. Endocrinology.

[B53] Blanco- Rodriguez J, Martinez- Garcia C (1998). Apoptosis precedes detachment of germ cells from the seminiferous epithelium after hormonal suppression by short-term oestradiol treatment of rats. Int J Androl.

[B54] Ellis GB, Desjardins C (1982). Male rats secrete luteinizing hormone and testosterone episodically. Endocrinology.

[B55] Bernstam L, Nriagu J (2000). Molecular aspects of arsenic stress. J Toxicol Environ Halth Prt- b- Critl Rev.

[B56] Kamel FA, Kubajak CL (1987). Modulation of gonadotrophin secretion by corticosterone interaction with gonadal steroids and mechanism of action. Endocrinology.

[B57] Bombino TH, Hsueh AJW (1989). Direct effect of glucocorticoids upon testicular luteinizing hormone receptor and steroidogenesis *in vivo *and *in vitro*. Endocrinology.

[B58] Kaur R, Dhanuju CK, Kaur K (1999). Effect of dietary selenium on biochemical composition in rat testis. Ind J Exp Biol.

[B59] Srivastava S, Sing GB, Srivastava SP, Seth PK (1990). Testicular toxicity of di- n- butyl phthalate in adult rats: effect on marker enzymes of spermatogenesis. Ind J Exp Biol.

[B60] Srivastava P, Vijayan E (1996). Testicular lactate dehydrogenase and sorbitol dehydrogenase activity after intratesticular injection of dymorphin and morphin in male rats. Ind J Exp Biol.

[B61] Vijayan E, McCann SM (1978). Re-valuation of the role of catecholamines in control of gonadotrophin and prolactin release. Neuroendocrinology.

[B62] Matsumoto AM, Paulson CA, Hopper BR, Rebar RW, Bremner WJ (1983). Human chorionic gonadotrophin and testicular function: stimulation of testosterone, testosterone precursors, and sperm production despite high estradiol levels. J Clin Endocrinol Metab.

[B63] Matsumoto AM, Bremner WJ (1985). Stimulation of sperm production by human chorionic gonadotrophin after prolonged gonadotrophin suppression in normal men. J Androl.

[B64] Chatterjee S, Ray A, Ghosh S, Bhattercharya K, Pakrashi A, Deb C (1988). Effect of aldrin on spermatogenesis, plasma gonadotrophins and testosterone, and testicular testosterone in the rat. J Endocrinol.

[B65] Matsumoto AM, Bremner WJ (1989). Endocrine control of human spermatogenesis. J Steroid Biochem.

[B66] Matsumoto AM, Karpas AE, Bremer WJ (1986). Chronic human chorionic gonadotrophin administration in normal men: evidence that follicle- stimulating hormone is necessary for the maintenance of quantitatively normal spermatogenesis in man. J Clin Endocrinol Metab.

[B67] Russell LD, Malone JP, Karpas SL (1981). Morphological pattern elicited by agents affecting spermatogenesis by disruption of its hormonal stimulation. Tissue Cell.

[B68] Shupnik MA, Gharib SD, Chin WW (1988). Estrogen suppresses rat gonadotrophin gene transcription *in vivo*. Endocrinology.

[B69] Ramaswamy S (2005). Pubertal augmentation in juvenile Rhesus monkey testosterone production induced by invariant gonadotrophin stimulation is inhibited by estrogen. J Clin Endocrinol Metab.

[B70] O'Donnell L, Robertson KM, Jones ME, Simpson ER (2001). Estrogen and spermatogenesis. Endocrine Rev.

[B71] Billing H, Furuta I, River C, Tapanainen J, Parvinen M, Hsueh JW (1995). Apoptosis in testis germ cells: developmental changes in gonadotrophin dependence and localization to selective tubule stages. Endocrinology.

[B72] Henriksen E, Hakovirta H, Parvinen M (1995). Testosterone inhibits and induces apoptosis in rat seminiferous tubules in a stage specific manner: in-situ quantification in squash preparations after administration of ethane dimethen sulfonate. Endocrinology.

